# Supplementary material for: Prevention of Taste Alterations in Patients with Cancer Receiving Paclitaxel- or Oxaliplatin-Based Chemotherapy—A Pilot Trial of Cannabidiol
Source: Nutrients. 2023 Jul 1;15(13):3014. doi: 10.3390/nu15133014 (PMC10346642; doi:10.3390/nu15133014)
Supplement: Supplementary file 1 [file nutrients-15-03014-s001.zip › nutrients-2390427-supplementary.pdf]

Table S1. Data from sensory test from patients in the control group. Patients indicated on a VAS-scale how intense the taste was perceived. Samples were randomised at testing.

Patient ID	Chemotherapy cycle nr.	**Salt**		**Sweet**		**Umami**	
		Weak	Strong	Weak	Strong	Weak	Strong
Control-1	1	35	10	01	03	01	01
Control-1	2	100	94	12	30	01	02
Control-1	3	48	95	01	01	04	03
Control-1	4	13	00	01	01	78	20
Control-2	1	08	86	84	71	00	91
Control-2	2	94	95	89	99	00	97
Control-2	3	60	100	24	100	100	0
Control-2	4	23	81	14	85	00	90
Control-3	1	38	73	20	69	28	45
Control-3	2	32	86	72	99	82	45
Control-3	3	67	56	90	92	75	52
Control-3	4	54	55	70	91	62	76
Control-4	1	56	52	05	36	13	07
Control-4	2	79	92	50	08	60	82
Control-4	3	94	91	54	94	52	54
Control-4	4	NA	NA	NA	NA	NA	NA
Control-5	1	46	81	44	53	01	04
Control-5	2	53	96	92	96	00	59
Control-5	3	95	98	44	74	51	93
Control-5	4	94	41	94	96	49	98
Control-6	1	88	54	27	99	00	00
Control-6	2	51	84	04	52	16	31
Control-6	3	55	49	13	21	22	06
Control-6	4	22	65	07	14	06	08
Control-7	1	59	62	00	49	44	67
Control-7	2	54	60	00	49	49	34
Control-7	3	00	60	54	73	00	56
Control-7	4	20	80	49	71	92	84
Control-8	1	53	98	11	94	04	91
Control-8	2	86	72	18	20	28	17
Control-8	3	65	94	11	96	83	05
Control-8	4	07	90	12	92	18	80
Control-9	1	83	96	23	92	34	46
Control-9	2	52	81	01	90	33	53
Control-9	3	47	00	12	83	22	46
Control-9	4	46	94	44	71	34	45
Control-10	1	22	58	23	42	21	52
Control-10	2	77	49	50	49	36	24
Control-10	3	37	80	31	65	51	48
Control-10	4	58	42	23	23	21	59

Table S2. Data from sensory test from patient 1-10 in the intervention group. Patients indicated on a VAS-scale how intense the taste was perceived. Samples were randomised at testing.

Patient ID	Chemotherapy cycle nr.	**Salt** Weak	Strong	**Sweet** Weak	Strong	**Umami** Weak	Strong
Intervention-1	1	40	69	49	44	26	06
Intervention-1	2	63	72	11	35	02	12
Intervention-1	3	NA	NA	NA	NA	NA	NA
Intervention-1	4	NA	NA	NA	NA	NA	NA
Intervention-2	1	80	54	NA	28	11	15
Intervention-2	2	20	NA	NA	41	NA	55
Intervention-2	3	51	69	72	12	NA	49
Intervention-2	4	52	69	21	42	48	14
Intervention-3	1	55	77	34	75	18	49
Intervention-3	2	82	15	10	75	15	29
Intervention-3	3	NA	NA	NA	NA	NA	NA
Intervention-3	4	54	72	35	25	30	27
Intervention-4	1	39	88	25	98	66	52
Intervention-4	2	35	50	00	87	85	86
Intervention-4	3	NA	NA	NA	NA	NA	NA
Intervention-4	4	NA	NA	NA	NA	NA	NA
Intervention-5	1	45	15	22	28	00	02
Intervention-5	2	34	18	25	26	25	33
Intervention-5	3	39	64	59	30	50	47
Intervention-5	4	55	41	60	71	60	49
Intervention-6	1	70	83	07	84	44	73
Intervention-6	2	NA	NA	NA	NA	NA	NA
Intervention-6	3	59	89	05	14	28	67
Intervention-6	4	NA	NA	NA	NA	NA	NA
Intervention-7	1	67	78	81	58	48	55
Intervention-7	2	95	72	58	54	64	54
Intervention-7	3	75	70	65	65	64	56
Intervention-7	4	84	85	52	67	49	55
Intervention-8	1	NA	71	49	96	NA	NA
Intervention-8	2	45	55	NA	50	NA	50
Intervention-8	3	48	59	48	46	08	50
Intervention-8	4	69	75	41	11	65	00
Intervention-9	1	52	76	10	59	68	19
Intervention-9	2	84	83	54	18	NA	100
Intervention-9	3	95	100	86	98	68	61
Intervention-9	4	97	99	NA	85	72	49
Intervention-10	1	25	82	58	22	53	91
Intervention-10	2	49	67	05	94	04	85
Intervention-10	3	42	95	05	97	17	35
Intervention-10	4	78	82	03	83	29	33

Table S3**.** Data from sensory test from patient 11-20 in the intervention group. Patients indicated on a VAS-scale how intense the taste was perceived. Samples were randomised at testing.

Patient ID	Chemotherapy cycle nr.	**Salt**		**Sweet**		**Umami**	
		Weak	Strong	Weak	Strong	Weak	Strong
Intervention-11	1	34	78	00	70	43	55
Intervention-11	2	35	53	42	60	NA	56
Intervention-11	3	45	75	06	71	00	00
Intervention-11	4	NA	NA	NA	NA	NA	NA
Intervention-12	1	00	52	83	52	89	00
Intervention-12	2	92	98	57	52	13	03
Intervention-12	3	05	06	50	95	00	23
Intervention-12	4	NA	91	NA	55	48	56
Intervention-13	1	42	94	62	52	45	37
Intervention-13	2	62	67	50	79	29	21
Intervention-13	3	65	72	34	81	33	59
Intervention-13	4	44	56	31	74	23	68
Intervention-14	1	85	87	53	60	26	30
Intervention-14	2	56	86	55	59	61	19
Intervention-14	3	75	87	73	70	70	88
Intervention-14	4	39	79	68	60	20	36
Intervention-15	1	35	72	91	29	01	00
Intervention-15	2	04	01	87	35	01	04
Intervention-15	3	81	90	02	61	01	09
Intervention-15	4	09	85	01	85	01	44
Intervention-16	1	04	03	64	76	56	50
Intervention-16	2	NA	NA	NA	NA	NA	NA
Intervention-16	3	NA	NA	NA	NA	NA	NA
Intervention-16	4	NA	67	03	42	NA	NA
Intervention-17	1	59	75	01	87	00	00
Intervention-17	2	91	76	06	92	19	49
Intervention-17	3	NA	NA	NA	NA	NA	NA
Intervention-17	4	NA	NA	NA	NA	NA	NA
Intervention-18	1	10	47	11	61	00	00
Intervention-18	2	05	11	NA	15	NA	12
Intervention-18	3	NA	NA	NA	NA	NA	NA
Intervention-18	4	15	58	00	23	NA	19
Intervention-19	1	73	95	21	90	08	50
Intervention-19	2	95	01	96	96	56	03
Intervention-19	3	33	93	32	95	48	64
Intervention-19	4	96	57	90	95	69	29
Intervention-20	1	77	20	60	14	24	12
Intervention-20	2	48	25	04	51	27	09
Intervention-20	3	22	86	55	76	22	21
Intervention-20	4	NA	NA	NA	NA	NA	NA

Table S4. Data from sensory test from patient 21-22 in the intervention group. Patients indicated on a VAS-scale how intense the taste was perceived. Samples were randomised at testing.

Intervention-21	1	71	94	61	85	58	75
Intervention-21	2	75	83	56	78	28	62
Intervention-21	3	NA	NA	NA	NA	NA	NA
Intervention-21	4	59	81	79	61	60	58
Intervention-22	1	100	96	53	95	04	54
Intervention-22	2	97	85	01	70	48	62
Intervention-22	3	67	22	01	25	01	61
Intervention-22	4	36	70	50	39	52	00

The control group has 2.4% missing answers distributed equally between salt, sweet and umami. The intervention group has 21% missing answers distributed with 6.44% missing on salt, 6.82% missing on sweet and 7.77% missing on umami.
